# Removal of Divalent Cations from Produced Water and Its Impact on Rheological Properties and Proppant Settling Velocity

**DOI:** 10.3390/gels11030158

**Published:** 2025-02-22

**Authors:** Yanze Zhang, Wajid Ali, Hassan Dehghanpour

**Affiliations:** Department of Civil and Environmental Engineering, School of Mining and Petroleum Engineering, University of Alberta, Edmonton, Alberta T6G 2W2, Canada; yanze1@ualberta.ca (Y.Z.); wajid@ualberta.ca (W.A.)

**Keywords:** flowback and produced water, friction reducers, hydraulic fracturing, rheological properties, proppant carrying capacity

## Abstract

The petroleum industry seeks to optimize the reuse of flowback and produced water (FPW) in hydraulic fracturing to reduce environmental impacts and costs. This study investigates how controlling divalent cations in FPW influences its rheological properties and proppant carrying capacity, both of which are crucial for efficient fracturing. Synthetic FPW, modified to simulate treated and untreated conditions, was analyzed to determine the impact of gel-based additives such as anionic polyacrylamide-based friction reducers (FRs). Results indicate that removing divalent cations increases relaxation times from 0.12 s in untreated FPW to 1.00 s in a 1 gallon per thousand gallons (gpt) FR solution, demonstrating improved viscoelastic gel characteristics. However, these changes do not significantly increase proppant carrying capacity. Even with relaxation times increasing to 4.5 s at higher FR dosages (3 gpt), the treated FPW still does not achieve the relaxation time observed in FR solutions using deionized (DI) water, which remain above 10 s. The removal of divalent cations from FPW resulted in only minor changes to its shear viscosity, with a modest 15% increase that was not enough to significantly affect the settling velocity of the proppant. Thus, removal of divalent cations can positively influence rheological behavior; it does not necessarily improve proppant transport efficiency in hydraulic fracturing operations.

## 1. Introduction

The restricted flow of oil and gas in low-permeability formations, such as shale and tight sandstones, makes hydrocarbon extraction challenging [1]. Slickwater fracturing, a type of hydraulic fracturing, has emerged as a preferred method for this process. This method employs the high-pressure injection of a specialized fluid mixture into the wellbore. This mixture primarily consists of water and proppants, along with a variety of additives, including friction-reducing agents, surface-active agents, scale inhibitors, clay stabilization compounds, and biocides [2,3,4]. Friction reducers (FRs), often gel-based polymers such as anionic polyacrylamides, are crucial for modulating fluid properties by imparting rheology to enhance flow efficiency. While proppants keep the fractures open, the additives enhance fluid flow and mitigate bacterial obstructions [5,6]. The efficacy of slickwater fracturing in shale reservoirs can be attributed to its optimized viscosity, viscoelastic properties, and ability to create intricate fracture networks [7,8].

Despite the efficacy of hydraulic fracturing, it raises concerns regarding significant freshwater consumption and the generation of large volumes of flowback and produced water (FPW). In the US and Canada, typical fracturing fluids are composed of approximately 94% freshwater, 5% proppant, and 1% chemical additives, including gel-based agents such as anionic polyacrylamides to improve fluid properties. Related reports indicate that freshwater can constitute up to 99.8% of the total fluid [9,10]. The development of a single horizontal well may require between 3 to 6 million gallons of water, highlighting the resource-intensive nature of the process [11]. This leads to increasing challenges and costs in sourcing water from both underground and surface water sources [9,12]. After the hydraulic fracturing process, a reduction in pressure within the wellbore causes the injected fracturing fluid to return to the surface, mixed with formation brine. Typically, in the initial two to four weeks post-fracturing, as much as 60% of the fracturing fluid is recovered at the surface [13]. Moreover, as oil and gas are extracted, formation water containing potentially toxic chemicals and consistently high concentrations of total dissolved solids (TDS) also emerges, posing environmental and public health risks [14,15,16,17]. Typically, FPW management includes treating and transporting water to disposal wells [18,19,20]. Nevertheless, the expenses associated with wastewater transportation and disposal can reach up to USD 14 per barrel of water [9].

Utilizing FPW in hydraulic fracturing activities presents a practical approach to address environmental issues related to high freshwater usage and the substantial production of FPW [21]. Utilizing FPW not only diminishes the environmental footprint of hydraulic fracturing but also reduces the freshwater demand in the petroleum industry. Nonetheless, while these benefits are notable, a major obstacle in reusing FPW for fracturing operations arises from its impact on the performance of fracturing fluid mixtures, particularly their rheological characteristics. This challenge is primarily attributed to compatibility issues between the FRs and the complex chemical composition of FPW [22].

The compatibility of gel-based FR with FPW presents significant challenges in reusing FPW for slickwater preparation in hydraulic fracturing operations. High concentrations of TDS and organic compounds in produced water can adversely affect the properties and performance of FRs [23]. Specifically, an increase in polyvalent cations concentration in FPW can lead to the “salting-out” effect. In this process, cations surround polymer molecules, disrupting the gel-like network formation by preventing the polymer chains from properly unfolding and dispersing in water [24]. Polyvalent cations can induce FR curling and clumping when surpassing certain concentrations. Particularly notable are Ca^2+^, Mg^2+^, Fe^2+^, and Fe^3+^, which tend to form complexes with polymer carboxyl groups, leading to a bridging effect [25,26,27]. This effect is significant because it not only promotes aggregation among molecules but also potentially results in polymer precipitation [28,29,30,31]. Such aggregation compromises the viscoelastic properties crucial for effective proppant transport [32,33]. As FRs agglomerate, the elongational viscosity crucial for turbulence suppression diminishes. This results in increased turbulence within the wellbore, leading to diminished drag reduction efficiency. Consequently, the pressure drop increases, impairing the proppant-carrying capacity of the gel-modified fluid and hindering the effective transport of proppants into the fractures [1].

Extensive research has been conducted to explore the rheological properties and settling velocities of FR solutions, crucial for the effective transport of proppants in hydraulic fracturing operations. Malhotra and Sharma (2012) investigated the settling velocity of spherical particles in both unbounded and confined surfactant-based shear-thinning fluids [34]. Their findings indicated that an increase in fluid elasticity led to an increase in drag. Another study investigating high-viscosity friction reducers (HVFRs) have demonstrated that these substances exhibit superior elastic properties and higher relaxation times across various shear rate ranges compared to linear polymer solutions [35]. The enhanced elasticity and viscoelastic properties of these FRs potentially facilitates more efficient proppant suspension and distribution within hydraulic fractures, indicating their advantage in hydraulic fracturing applications. Numerous studies have similarly emphasized how the viscosity and elasticity of fluids play crucial roles in the transport of particles [36,37,38]. Conversely, a comprehensive analysis conducted by Galindo (2019), which examined 28 commercial products, suggests that gels’ viscosity might not be the primary factor influencing proppant transport [39]. This study found that low shear viscosity did not markedly differentiate product performance in sand-transport testing, challenging the conventional emphasis on high viscosity as a key factor in proppant-transport efficiency.

Despite these advancements, the literature reveals a significant gap in studies specifically addressing the reuse of FPW in such applications. There is a lack of detailed investigations examining how modifications to the composition of FPW, primarily through the removal of divalent cations, affect its shear viscosity and viscoelastic properties. Furthermore, the impact of these altered properties on proppant carrying capacity has not been adequately explored. Our primary objective is to enhance the operational feasibility of using FPW in hydraulic fracturing by systematically studying the influence of divalent cation removal on the rheological behavior of slickwater prepared from FPW. We aim to understand how these changes affect the ability of fluids to carry proppants, providing insights that could lead to more effective and environmentally friendly hydraulic fracturing practices.

## 2. Experimental Results and Discussions

To evaluate the effectiveness of divalent cation removal, the FPW chemical composition was analyzed before and after chemical softening. Further, the rheological properties of the FR solutions were determined to assess the effect of divalent cation removal on shear viscosity and viscoelasticity. Lastly, the settling velocity of the sand proppants in the FR solution was measured to evaluate the effectiveness of the treatment in enhancing proppant suspension and distribution within the FPW samples. The combination of these three studies provides a comprehensive assessment of the treatment efficacy and its potential impact on hydraulic fracturing operations.

### 2.1. Optimization of FPW Treatment

In this study, we directly demonstrated the superior efficacy of the proposed treatment, which was validated through our own preliminary tests and detailed simulations using PHREEQC Interactive 3.7.3 software. Using PHREEQC is beneficial as it allows for the accurate prediction of chemical interactions under various conditions, reducing the need for extensive physical experimentation. This approach involved calculated doses of Na_2_CO_3_ and NaOH, which effectively reduced concentrations of divalent cations without altering the overall composition of the FPW.

Experimental trials were conducted by varying the molar ratio of Na_2_CO_3_ to the initial total concentration of divalent cations between 0.8 and 1.2. A molar ratio of “1.0” was designated as the equimolar dose, provided stoichiometric equilibrium with divalent cations. To further enhance the removal efficiency of divalent cations, varying doses of NaOH were added incrementally after the equimolar addition of Na_2_CO_3_. This stepwise addition was intended to optimize cation removal without excessively altering the chemical balance. The addition of these alkaline compounds resulted in an increase in the pH of the solution, reflecting their inherent properties to elevate pH levels. The concentrations of divalent cations in the post-treatment solution, denoted as C_Ca_, C_Mg_, C_Fe_ and C_Sr_, respectively, were determined using ICP-OES analyses and are detailed in Table 1. Correspondingly, the removal efficiencies of these cations were recorded as E_Ca_, E_Mg_, E_Fe_, and E_Sr_.

The results presented in Table 1 illustrate trends in divalent cation removal efficiencies and accompanying pH variations resulting from varied Na_2_CO_3_ and NaOH dosages during FPW treatment. A discernible trend shows removal efficiencies for Ca^2+^, Mg^2+^, Sr^2+^, and Fe^2+^ as the molar ratio of Na_2_CO_3_ increases from 0.8 to 1.2. This pattern indicates that higher Na_2_CO_3_ doses are more effective at enhancing divalent cation removal through the precipitation reactions during the treatment process [40,41,42]. The inclusion of Fe^3+^ in the following equations is based on the observed oxidation of Fe^2+^ to Fe^3+^ when exposed to air, a reaction that was consistently noted as the synthetic brine transitioned to a yellowish color, similar to conditions found in real flowback produced water.

(1) $${Ca}_{\left(aq\right)}^{2+}+{CO}_{3\;(aq)}^{2-}↔Ca{CO}_{3(s)}\;\;\;\;\;\;\;\;\;logK=8.12$$

(2) $${Ca}_{\left(aq\right)}^{2+}+{Mg}_{\left(aq\right)}^{2+}+{2CO}_{3\;\left(aq\right)}^{2-}\;↔CaMg({CO}_{3}{)}_{2\left(s\right)}\;\;logK=17.41$$

(3) $${Fe}_{\left(aq\right)}^{3+}+3{OH}_{\left(aq\right)}^{-}\;↔{Fe(OH)}_{3(s)}\;\;\;\;\;\;\;\;logK=38.52$$

(4) $${Sr}_{\left(aq\right)}^{2+}+{CO}_{3\;(aq)}^{2-}↔Sr{CO}_{3(s)}\;\;\;\;\;\;\;\;\;logK=2.81$$

However, when the dosage of Na_2_CO_3_ was increased to 1.2 times the equimolar concentration, C_Mg_ decreased to 169.2 ppm. This observation is consistent with research findings by Liu et al. (2020), which indicated that Mg^2+^ ions cannot be completely removed using Na_2_CO_3_ alone [43]. The addition of NaOH, especially at equimolar levels with Na_2_CO_3_, significantly enhanced Mg^2+^ removal efficiencies, indicating a synergistic interaction between these compounds. The improved removal of Mg^2+^ is attributed to the following precipitation reactions that occur upon adding NaOH to FPW.

(5) $${{Mg}_{\left(aq\right)}^{2+}+{2OH}_{\left(aq\right)}^{-}↔Mg(OH)}_{2(s)}\;\;\;logK=11.25$$

Among the tested Na_2_CO_3_ and NaOH concentrations for FPW treatment, optimal divalent cation removal was achieved using a combination of equimolar Na_2_CO_3_ and 0.0167 mol/L NaOH.

### 2.2. Rheological Properties Evaluation

Here, we evaluated the viscosity and viscoelastic properties of FR solutions prepared from three different sources: untreated FPW (UTFPW), and treated FPW (TFPW), and DI water. This comparative analysis allowed us to assess the impact of removing divalent cations such as Ca^2+^, Mg^2+^, Sr^2+^, and Fe^2+^ on the rheological behavior of FR solutions. Tests were conducted across varying FR concentrations of 1 gpt, 2 gpt, and 3 gpt to further explore these effects.

#### 2.2.1. Shear Viscosity of FR Solutions

**Figure 1:**
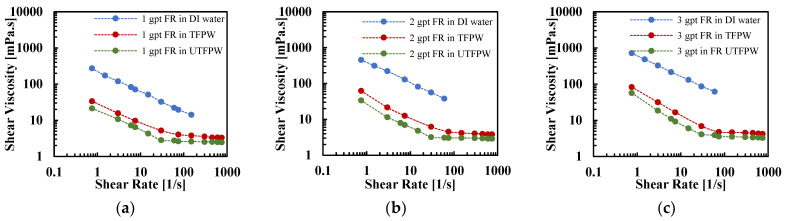
Comparative analysis of shear viscosity profiles across different aqueous media: FR solutions at incremental concentrations of (**a**) 1 gpt, (**b**) 2 gpt, (**c**) 3 gpt in DI water, TFPW, and UTFPW.

The FR solution exhibited shear-thinning behavior in DI water, UTFPW, and TFPW, as depicted in Figure 1 with rheological parameters obtained from curve fitting shown in Table 2. The FR in DI water exhibited a near-ideal shear-thinning behavior, with viscosity decreasing exponentially as shear rate increased. When the FR concentration in DI water was increased from 1 gpt to 3 gpt, the flow consistency index (*K*) value showed significant improvements: an increase of 76.8% at 2 gpt and 169.2% at 3 gpt compared to the baseline at 1 gpt. These elevated *K* values indicate enhanced fluid viscosity, which correlates directly with increasing FR concentration. This trend is consistent with the expected behavior of polymeric additives, where higher concentrations lead to an increase in interactions among polymer chains, thus significantly enhancing the fluid’s resistance to flow. In contrast, in UTFPW, the power-law model was inadequate due to the presence of a second constant viscosity region, denoted as $\eta_{\infty }$, observed in these solutions. Instead, the Sisko model proved more appropriate for characterizing the rheological behavior.

The shear viscosity curves in UTFPW experienced a sharper decline with increasing shear rate compared to those in DI water, which is also reflected by the lower values of the flow behavior index *n*, as presented in Table 2. As the concentration of FR increased in UTFPW, the rise in shear viscosity was minimal. The K values indicated only a modest improvement of 13.64% when the concentration increased from 1 gpt to 2 gpt, and a more substantial improvement of 81.82% at 3 gpt compared to the 1 gpt baseline. However, the *K* values in UTFPW remain relatively low compared to those in DI water. This limited response is due to FR particle agglomeration, influenced by the presence of multivalent cations, particularly divalent cations [44,45].

Notably, both *K* and *n* values are higher in TFPW compared to UTFPW at equivalent FR concentrations, suggesting a more pronounced shear-thinning behavior in TFPW. Specifically, the improvement in *K* value is 22.73% at 1 gpt, 88% at 2 gpt, and 65% at 3 gpt, indicating that the treatment process enhances the rheological properties of the water due to the removal of divalent cations. Despite these improvements, the *K* values in both UTFPW and TFPW remain substantially lower compared to those in DI water. In FR solutions prepared with UTFPW, the shear-thinning behavior is pronounced, as indicated by significantly reduced *n* values at higher shear rates. However, increasing the FR concentration does not proportionally enhance this behavior, as seen with 1 gpt FR in TFPW having an *n* value of 0.2533 compared to a lower *n* value of 0.0753 at 3 gpt.

Moreover, $\eta_{\infty }$ provides additional insights into the fluid behavior at different shear rates. For FR solutions in UTFPW, $\eta_{\infty }$ is reached at around 15 1/s, indicating a quicker response to shear stress. In contrast, for TFPW, this viscosity level is attained at a higher shear rate of approximately 80 1/s. As the FR concentration increases, there is a slight increase for $\eta_{\infty }$ value for both UTFPW and TFPW.

#### 2.2.2. Viscoelasticity of FR Solutions

**Figure 2:**
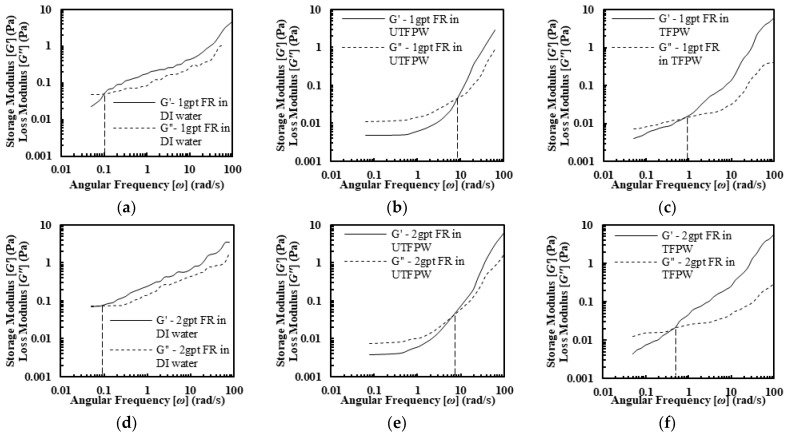

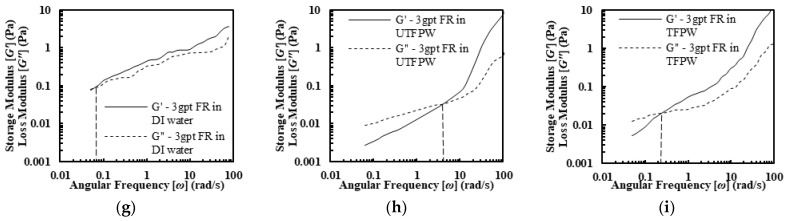
Viscoelastic profiles (*G*′ and *G*″) of FR solutions in (**a**,**d**,**g**) DI water, (**b**,**e**,**h**) UTFPW, and (**c**,**f**,**i**) TFPW with FR concentration varying from 1 gpt to 3 gpt.

The viscoelastic properties of FR solutions exhibit distinct patterns across different water types and FR concentrations, as shown in Figure 2, with DI water solutions demonstrating consistently higher storage modulus (*G*′) and loss modulus (*G*″) values and increasing relaxation times (*λ*) from 10 s to 15.4 s as concentration rises from 1 to 3 gpt. As summarized in Table 3, UTFPW solutions show similar viscoelastic profiles to DI water at higher frequencies but *G*′ and *G*″ decrease significantly at lower frequencies, maintaining relatively stable *λ* values between 0.12 and 0.25 s across FR concentrations. When divalent cations are removed from FPW, the viscoelastic properties show improvement compared to those in UTFPW. The *λ* increase from 1.11 s at 1 gpt to 4.55 s at 3 gpt. However, these values remain lower than those observed in DI water solutions. This is likely due to the high NaCl concentration, which interferes with FR interactions.

### 2.3. Settling Velocity Evaluation

The study examines how removing divalent cations from FPW affects proppant settling behavior and FR solution properties. As reported in Table 4, settling velocities decreases as FR concentrations increases from 1 gpt to 3 gpt across all proppant sizes and types of fluids, demonstrating improved proppant suspension at higher FR concentrations. The data also showed that smaller proppants (40–70 mesh) consistently settle more slowly than larger ones (16–30 mesh) due to their higher surface area to volume ratio, which increases fluid drag resistance against gravitational forces [46]. Proppants in UTFPW consistently show higher settling velocities compared to those in DI water and TFPW at corresponding FR concentrations and proppant sizes. The settling velocities in TFPW are slightly lower than those in UTFPW but still higher than those in DI water. This indicates an insignificant improvement in the proppant settling velocity when the divalent cations are removed from FPW.

#### 2.3.1. Role of Shear Viscosity Post-Divalent Cation Removal

In this analysis, we have adjusted the particle Reynolds numbers (*Re_p_*) to account for the shear-thinning behavior observed in the fluid dynamics and compared the resulting drag coefficient (*C*_*D*_) with those from the universal curve for spheres in Newtonian fluids. The *C*_*D*_ values were calculated based on experimentally measured settling velocities as listed in Table 4.

**Figure 3:**
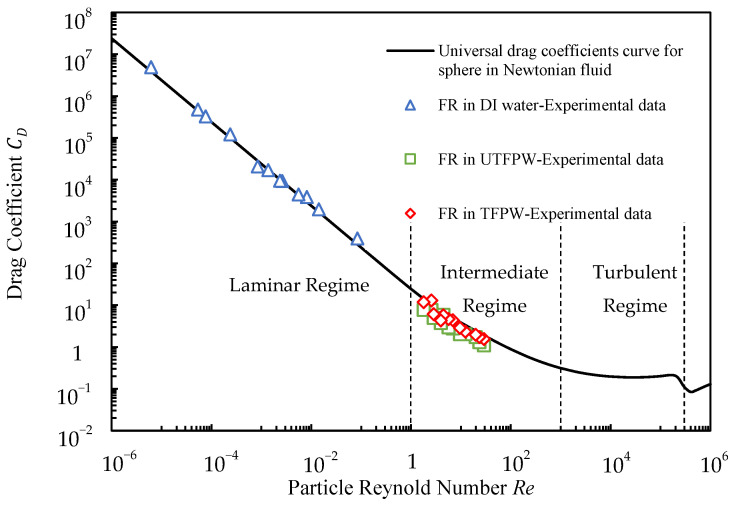
Comparison of drag coefficients and modified Reynolds numbers for proppants in shear-thinning FR solutions with universal values for spheres in Newtonian fluids.

Figure 3 demonstrates that the modified data closely align with the universal drag coefficients (C_D*_) curve. Proppants of all sizes suspended in FR solutions formulated with DI water exhibit laminar flow patterns around the sphere, consistently characterized by *Re_p_* below 2. In contrast, proppants in UTFPW and TFPW displayed flow regimes in the intermediate regime. Notably, the behavior of proppants in FR solutions prepared with both UTFPW and TFPW appears nearly identical, indicating minimal enhancement in proppant carrying capacity despite the removal of divalent cations from FPW.

**Figure 4:**
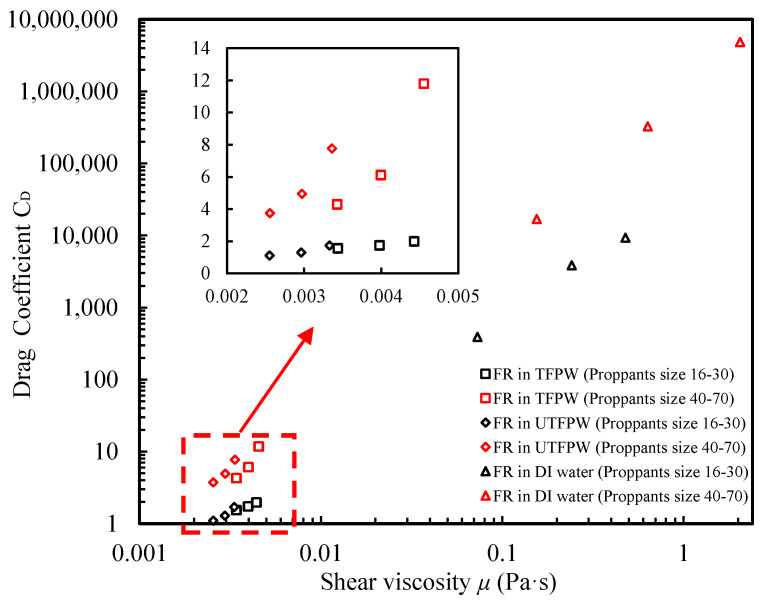
Comparison of drag coefficients versus shear viscosity for proppants of various sizes settled in different fluids. Shear viscosity is derived from calculated shear rates using Equations (13) and (14).

Figure 4 illustrates the relationship between *C*_*D*_ and fluid’s viscosity (*μ*) for proppants settling in three different fluid types. It is evident that *C*_*D*_, which significantly influences the settling velocity, is directly affected by *μ* of the fluid. In these experiments, variations in shear viscosity arise from differing concentrations of FR in the solutions. In DI water, proppants experience a notably higher *C*_*D*_, especially as particle size decreases, indicating that smaller particles encounter greater drag compared to larger particles in higher *μ* environments. When proppants are suspended in UTFPW prepared with FR solution, they exhibit the lowest drag, resulting in minimal settling velocities. However, after the removal of divalent cations from FPW to prepare the FR solution, *C*_*D*_ increases slightly compared to UTFPW but remains substantially lower than that of DI water.

Despite the removal of divalent cations, no significant improvement in *μ* was observed, resulting in relatively low *C*_*D*_ values. These findings align with Arnipally and Kuru’s (2018) research, which demonstrated that settling velocity decreases as shear viscosity increases [46]. Additionally, the data revealed a correlation where higher *K* values, which indicate increased fluid viscosity, are associated with a decrease in the settling velocity of particles. Our findings further validate these observations by demonstrating that settling velocity is significantly influenced by both *K* and *n* values. Despite the removal of divalent cations from FPW, which theoretically should reduce the settling velocity and improve the fluid’s ability to suspend particles, no significant reduction in settling velocity was observed. This phenomenon can be attributed to the low *n* values observed, indicating that *μ* decreases considerably at higher $\dot{\gamma }$, ultimately affecting the settling dynamics.

#### 2.3.2. Role of Viscoelasticity Post-Divalent Cation Removal

**Figure 5:**
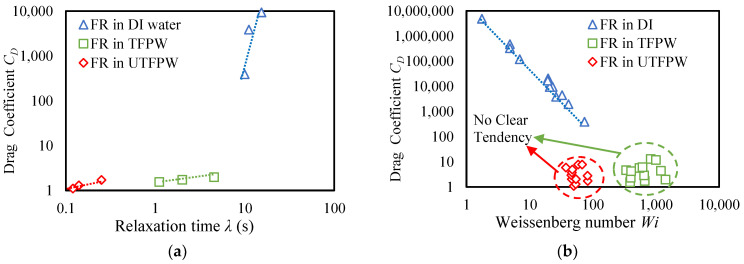
Correlations between calculated drag coefficient across different fluid types: (**a**) with λ for proppant size 16–30, and (**b**) with Weissenberg number obtained from shear viscosity and viscoelasticity measurements.

Figure 5a illustrates the relationship between *C_D_* and *λ* for proppants of size 16–30 in three different fluid types: DI water, UTFPW, and TFPW. FR solutions prepared with DI water exhibit high *C_D_* values, even though their *λ* values do not substantially increase with higher FR concentrations, remaining at 10 s at 1 gpt and only increasing to 15.4 s at 3 gpt, as shown in Table 3. This observation suggests that the high *λ* indicative of significant viscoelasticity in DI water play a crucial role in the observed increase in drag coefficient. Additionally, even a slight increase in *λ* can substantially elevate the drag coefficient, highlighting the sensitivity of drag to changes in viscoelastic properties. In contrast, FR solutions in UTFPW show the lowest *C_D_* and *λ* values among the three fluid types. Although *λ* in TFPW is substantially increased compared to UTFPW, their *C_D_* values are very similar. This suggests that the dependency of the drag coefficient on viscoelastic properties in UTFPW is weak. Similarly, for TFPW, despite the removal of divalent cations, which increases viscoelasticity and hence recovers *λ*, there is no corresponding rise in *C_D_*. 

For the FR solution made with DI water, as the Weissenberg number (*W_i_*) increases, *C_D_* also increases significantly compared to the other two fluid types, as shown in Figure 5b. This suggests that particles in DI water experience greater fluid resistance, due to the higher viscosity of the FR solution. In UTFPW, *C_D_* remains relatively low across the range of *W_i_*, especially when compared to the higher *C_D_* values observed in FR solutions made with DI water and TFPW. 

For FR in TFPW, the observed data indicate that *C_D_* stays relatively low, even as *W_i_* increases, with only a negligible effect even as *W_i_* values increase. The elevated *W_i_* is attributed to increased *λ* and higher $\dot{\gamma }$. Despite the increased viscoelastic properties resulting from the removal of divalent cations, this improvement exerts no significant influence on the fluid’s resistance when subjected to higher $\dot{\gamma }$. This observation suggests that while the treatment increases the fluid’s viscoelastic characteristics, its efficacy in increase drag under conditions of high shear is limited. He et al. (2018) analyzed particle settling in crosslinked carboxymethyl hydroxypropyl guar (CMHPG) and concluded that delayed settling is influenced by both increased viscosity and elastic lifting forces. They determined that the relative importance of these factors varies with $\dot{\gamma }$ and fluid *λ* [47]. Specifically, they observed that shear viscosity is more influential at shear rates below 2 s^−1^, whereas elasticity predominates at rates above 2 s^−1^. Similarly, Zhang et al. (2016) discovered that the settling velocity of proppant particles increases by increasing *W_i_* [48]. Our findings here seem to indicate that high concentrations of Na^+^ can also hinder proppant settling due to significant shielding effects, which particularly influence the shear viscosity of the fluid. Although removing divalent cations mitigates the bridging effect, the shielding effect induced by Na^+^ remains a critical factor affecting fluid dynamics [49,50].

## 3. Conclusions 

In this study, we have analyzed the rheological properties and proppant carrying capacity of FR solutions formulated with flowback and produced water (FPW). The primary goal was to assess the effectiveness of removing divalent cations from FPW in improving its suitability for hydraulic fracturing operations. This objective was achieved through a two-fold investigation: firstly, assessing whether the removal of divalent cations could improve the shear viscosity and viscoelasticity of the FPW; and secondly, evaluating if these enhanced rheological properties could in turn increase the proppant carrying capacity.

1. The FR solutions in DI water, untreated FPW (UTFPW), and treated FPW (TFPW) all displayed shear-thinning behavior. In DI water, FR solutions with different concentrations exhibited near-ideal shear-thinning with viscosity decreasing exponentially with increasing shear rates. In contrast, the power-law model was not suitable for UTFPW due to the presence of a second constant viscosity ($\eta_{\infty }$,), and the Sisko model provided a better fit. In UTFPW and TFPW, shear viscosity sharply declined despite increased FR concentration, which was accompanied by low flow behavior index (*n*) values and flow consistency index (*K*). Meanwhile, removing divalent cations from FPW resulted in increased *K* and *n* values. However, this treatment failed to maintain viscosity at high shear rates compared to solutions with DI water. FR solutions prepared with DI water exhibit significantly higher viscoelastic properties compared to those made with UTFPW and TFPW. Additionally, relaxation times (λ) increase with higher concentrations, further outperforming those made with UTFPW and TFPW.
2. The removal of divalent cations from FPW increases the relaxation time (λ) from 0.12 s to 1 s at a 1 gpt concentration of FR. Further increases in FR concentration raise λ to 4.5 s. However, the high NaCl content constrains these improvements, keeping them below the levels achieved with DI water.
3. Although the removal of divalent cations increases *λ* of the fluids, this increase in *λ* does not significantly increase the settling velocity of the proppants compared to the results observed with FR in UTFPW. The drag coefficient shows a dependency on shear viscosity, remaining high when viscosity is elevated and diminishing as viscosity decreases. Furthermore, despite the improved viscoelastic properties resulting from the removal of divalent cations, these changes do not contribute to an increase in proppant carrying capacity due to the fluid’s persistently low shear resistance. This persistently low viscosity can be attributed primarily to the high NaCl concentration, which may inhibit the effective increase in viscosity even after the removal of divalent cations.

### Limitation and Future Work

4. A key limitation of this study is its reliance on controlled laboratory conditions, which may not fully represent real-world applications. The removal efficiency of divalent cations and rheological measurements were conducted under standardized laboratory settings, without accounting for the variable temperature, pressure, and fluid dynamics encountered in field operations. The controlled range of FR concentrations (1–3 gpt) and the use of a confined cylinder setup for settling velocity experiments may not adequately capture the complex proppant transport behavior within actual fracture geometries, particularly in horizontal wellbores.
5. Future research should concentrate on investigating the combined effects of monovalent and divalent cations on the performance of FRs. Understanding how these cations interact can help optimize salinity levels for more efficient hydraulic fracturing operations.
6. Additionally, it is crucial to evaluate how temperature-dependent changes in viscoelastic properties influence FR behavior. This aspect of research is vital for tailoring fluid formulations to better withstand the thermal variations encountered during hydraulic fracturing.

## 4. Materials and Methodology

### 4.1. Material

Our study presents the detailed chemical composition of real FPW samples obtained from hydraulic fracturing operations in the Duvernay Formation, a shale play located in the Fox Creek area of Alberta, Western Canada [51]. We also highlight the chemical additives, specifically anhydrous Na_2_CO_3_ and NaOH, used for the precipitation and removal of divalent cations, including Ca^2+^, Mg^2+^, Sr^2+^, and Fe^2+^ from the FPW samples. Furthermore, we provide a summary of the key properties of the FR sample used in our experiments.

#### 4.1.1. Flowback and Produced Water (FPW)

The sample contains approximately 551 mg/L total organic carbon (TOC) and 8580 mg/L total suspended solids (TSS). Table 5 provides a detailed breakdown of the cation and anion contents in the FPW sample.

#### 4.1.2. Chemical Reagents

We used anhydrous sodium carbonate (Na_2_CO_3_ > 99.5% purity, supplied by Fisher Scientific, Pittsburgh, PA, USA), recognized for its effectiveness in precipitating Ca^2+^, Mg^2+^, Sr^2+^, and Fe^2+^ ions. As demonstrated by Wang et al. [40], utilizing Na_2_CO_3_ can precipitate approximately 85% of Ca^2+^ from seawater when processed at temperature of 85 °C and a pH of 11. Moreover, Na_2_CO_3_ offers the advantage of removing divalent cations without inducing any side reactions that could alter the composition of the FPW sample. Analytical-grade sodium hydroxide (NaOH > 97.0% purity, supplied by Fisher Scientific) was employed to adjust the pH of the FPW sample, enabling precise control at targeted levels and supporting the primary objective of reducing divalent cation concentrations.

Additionally, the synthetic FPW was prepared using sodium chloride (NaCl > 99% purity), sodium bicarbonate (NaHCO_3_; >99% purity), magnesium chloride hexahydrate (MgCl_2_·6H_2_O; >99% purity), calcium chloride Dihydrate (CaCl_2_·2H_2_O; 99% purity), ferrous sulfate heptahydrate (FeSO_4_·7H_2_O; >99% purity), strontium chloride hexahydrate (SrCl_2_·6H_2_O; 99% purity) and deionized water (DI). All chemicals were supplied by Fisher Scientific with the composition of the synthetic FPW comparable to the values presented in Table 5.

#### 4.1.3. Friction Reducer Additive

This study evaluates the efficacy of treating FPW prior to its use as a fracturing fluid. For this investigation, an emulsified anionic polyacrylamide (EPAM) gel-based formulation was chosen as the FR. The EPAM sample used in this study contains a polymer concentration of 35%, carried by mineral oil. This formulation has a specific gravity of 1.05 and a variable charge density, optimized to the needs of hydraulic fracturing applications. The EPAM’s polyacrylamide component has a molecular weight in the range of 10–20 million Da. These properties enhance the FR’s ability to modify fluid viscosity and improve proppant-carrying capacity, making it highly suitable for hydraulic fracturing operations.

#### 4.1.4. Proppants

In this study, four distinct sand proppant sizes were employed: 16/30, 20/40, 30/50, and 40/70. These designations refer to standard sieve cuts commonly used to classify the size of frac sand particles. For instance, a 16/30 proppant indicates that the sand particles pass through a 16-mesh sieve but are retained by a 30-mesh sieve, meaning the particle sizes fall within the range defined by the openings of the 16 and 30 mesh screens. Detailed information regarding the properties of these proppants is provided in Table 6. The sphericity of these proppants was estimated through visual assessments and comparisons with standard morphological descriptions found in the literature, which are commonly widely accepted for classifying particle shapes in such materials [52,53].

### 4.2. Methodology

In this section, we describe the experimental methodology used to assess the feasibility of using treated FPW, with divalent cations removed, for potential application in hydraulic fracturing operations. The study comprises two main experimental sections: Firstly, we optimized the treatment of FPW for divalent cation removal (Ca^2+^, Mg^2+^, Sr^2+^, and Fe^2+^) while minimally altering its composition, assessed via inductively coupled plasma-optical emission spectrometry (ICP-OES). Secondly, we evaluated the rheological properties and proppant settling velocities of fracturing fluids prepared with treated FPW, measuring shear viscosity and viscoelasticity to determine their efficacy in maintaining proppant suspension.

#### 4.2.1. Chemical Treatment of FPW

We evaluated the effectiveness of removing Ca^2+^, Mg^2+^, Sr^2+^, and Fe^2+^, ions from FPW by varying the dosages of NaOH and Na_2_CO_3_. Our experimental strategies aimed to optimize the removal efficiency of these divalent cations while avoiding the introduction of additional cation types and significant pH alterations. To achieve this, we employed the PHREEQC, which facilitated the identification of optimal treatment conditions.

A known sample of FPW was added to a glass container, which was then placed on a magnetic stirrer. Powdered NaOH was gradually added to adjust the pH. The sample’s pH and temperature were continuously monitored using a digital pH meter and a glass thermometer. Calculated dosages of solid Na_2_CO_3_, ranging from 0.8 to 1.2 eq/mol based on the initial divalent cation’s concentrations, were introduced into the FPW sample, and the mixture was stirred at 200 rpm for 45 min. After stirring, the sample was allowed to settle for 6 h to enable suspended particles and precipitates to settle to the bottom of the container. After the treatment, all treated samples were filtered using a 0.45 µm cellulose acetate filter to remove any remaining suspension and precipitate before preparing the FR solutions.

#### 4.2.2. Water Chemistry Analysis

The concentrations of Na^+^, Ca^2+^, Mg^2+^, Sr^2+^, and Fe^2+^ ions in the TFPW samples were determined using the Thermo iCAP6300 Duo ICP-OES instrument (supplied by Thermo Fisher Scientific, Waltham, MA, USA). To evaluate the efficiency of the chemical treatment, the concentrations of the divalent cations (Ca^2+^, Mg^2+^, Sr^2+^, and Fe^2+^) were measured and compared before and after the treatment process. Additionally, a portable pH meter manufactured by Hanna instruments was employed to monitor the pH regularly throughout the experiments.

#### 4.2.3. FR Solution Preparation

The FR solutions were prepared using DI water, UTFPW, and TFPW. Initially, a measured volume of each water sample was stirred at 400 rpm overnight to remove any entrapped air bubbles, ensuring optimal conditions for the formation of gel-like structures. Subsequently, a precise amount of FR was added to each sample using a calibrated sampler to prepare solutions with concentrations of 1 gallon per thousand gallons (gpt), 2 gpt, and 3 gpt. These concentration levels are well-established in the literature and field applications as optimal for achieving the desired performance and efficiency of the fracturing fluid [22,54,55]. For reference, 1 gpt corresponds to adding 1 milliliter of FR per liter of solution (mL/L). The prepared solutions were then stirred at 500 rpm for five minutes, as the dissolution time for emulsion-based FR typically ranges from 5 to 10 min. After this initial stirring, the speed was reduced to 100 rpm and was maintained until the polymer had completely dissolved. Finally, a 45 min rest period was given to ensure full dissolution of the FR and stabilization of the solution’s properties [1].

#### 4.2.4. Shear Viscosity Measurements

Shear viscosity measurements are critical to monitor and control the friction reduction and proppant-carrying ability of the FR solution [56,57]. Maintaining a specific shear viscosity is essential for achieving laminar flow conditions, which are crucial for the optimal performance of fracturing fluids. Laminar flow minimizes energy losses caused by turbulence and ensures the consistent and controlled delivery of fluid throughout the wellbore and fractures [2].

The shear viscosity of the FR solutions was measured at room temperature using a Brookfield cone/plate viscometer. Specifically, we utilized a cone with a diameter of 2.4 cm and a cone angle of 0.8 degrees. The shear viscosity profiles were obtained by measuring shear stress and apparent viscosity across shear rates ranging from 0.1 to 100 s^−1^ using a rotational rheometer. In general, polymer solution behavior can be described by the power law model [58]:

(6) $$\tau =K{\dot{\gamma }}^{n}$$

The power-law model employs *K* (Pa·s^*n*^) and *n* to characterize non-Newtonian fluids based on the relationship between shear stress and shear rate. In this model, *τ* denotes the shear stress (Pa), *μ* represents the fluid’s viscosity (Pa·s), and $\dot{\gamma }$ signifies shear rate (1/s). Typically, when *n* = 1, the fluid behaves as a Newtonian fluid, whereas a lower *n* indicates increased shear-thinning behavior. The *K* and *n* values for all fluids in this study were determined by curve fitting the measured shear stress and shear rate data.

Although the power-law model is widely used to describe the behavior of many non-Newtonian fluids, it does not fully capture the fluid dynamics under certain conditions, particularly at higher shear rates or stresses. In such cases, the Sisko model [59] provides a more accurate representation. This model accounts for the flattening of the viscosity curve at high shear rates and, with sufficient data from these conditions, often reveals the presence of $\eta_{\infty }$. $\eta_{\infty }$ represents a region where the fluid achieves a stable viscosity level despite further increases in shear, indicating that the fluid’s molecular structures have reached a state of maximum alignment and minimal interaction.

(7) $$\tau =\eta_{\infty }\dot{\gamma }+K{\dot{\gamma }}^{n}$$

#### 4.2.5. Viscoelasticity Measurements

The capability of FR solutions to carry proppants is significantly influenced by their viscoelastic properties [60,61]. It is important to measure these viscoelastic properties because they can help reduce the frictional resistance, ensure a laminar flow, and maintain uniform suspension of proppant. Arnipally and Kuru [46] investigated the relative importance of viscoelasticity and shear viscosity in controlling particle settling velocity in viscoelastic drilling fluids. Their findings indicated that while both fluid shear viscosity and elasticity substantially affect particle-settling velocity, high shear viscosity is not always practical due to the increase in parasitic pressure losses it induces. Consequently, enhancing fluid elasticity might reduce particle settling velocity even with lower shear viscosity, which is particularly advantageous for transporting large-sized drill cuttings.

We measured the viscoelastic properties of the FR solutions by using a TA Discovery HR-3 Rheometer. This advanced instrument is equipped with a 40 mm diameter stainless steel parallel plate setup, featuring a Peltier plate for precise temperature control, ensuring consistent test conditions. For all measurements, the strain amplitude was set at 1%, which falls within the linear viscoelastic region (LVR). The viscoelastic properties were further characterized by assessing the intersection value of the *G*′ and *G*″ as a function of angular frequency (*ω*) [62]. This analysis highlights how *G*′ and *G*″ respond to changes in deformation speed, with *G*′ reflecting the material’s capacity to store energy elastically during rapid cyclic deformations and *G*″ indicating the extent of energy dissipation through internal friction and viscous flow. The relationship between the *G*′ and the *G*″ is quantitatively expressed as the inverse of the product of ω and λ, given by the following equation [63]:

(8) $$G^{\prime }=\frac{G{\left(\omega \lambda \right)}^{2}}{1+{\left(\omega \lambda \right)}^{2}}$$

(9) $$G^{\prime \prime }=\frac{G\left(\omega \lambda \right)}{1+{\left(\omega \lambda \right)}^{2}}$$

A scenario where *G*′ is less than *G*″(*G*′(*ω*)/*G*″(*ω*) < 1) indicates a predominance of viscous behavior, suggesting weaker intermolecular interactions within the polymer matrix. In contrast, when *G*′ exceeds *G*″(*G*′(*ω*)/*G*″(*ω*) > 1), it reflects a more pronounced elastic behavior, implying stronger intermolecular forces within the polymer network [64,65]. At the crossover point where *G*′(*ω*)/*G*″(*ω*) = 1, *ω* is inversely proportional to λ of the system. A longer λ correlates with a fluid’s increased ability to suspend proppants [61].

The tests were conducted under ambient conditions, with the ω range for all measurements set between 0.05 and 100 rad/s. These measurements offer insights into how removing divalent cations from FPW can enhance the viscoelastic properties of FR solutions, thereby improving their ability to carry proppants [66].

#### 4.2.6. Settling Velocity Measurements

**Figure 6:**
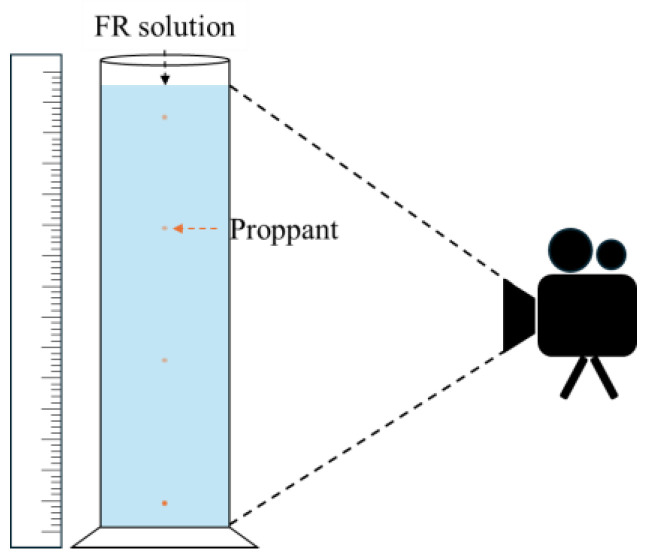
Schematic diagram of proppant settling dynamics in FR solutions.

Experiments were conducted using a transparent cylinder measuring 34.5 cm in height and 4.8 cm in diameter filled with FR solution. Initially, proppants were individually immersed in the fluid within a graduated cylinder, ensuring the formation of a thin fluid layer around each particle to prevent air bubble formation and promote quick achievement of settling velocity. A schematic diagram is illustrated in Figure 6. This procedure was replicated 20 times for each proppant size across various fluid types. All experimental steps were meticulously recorded, and the “Tracker 6.2.0” software was employed to analyze and measure the settling velocities.

To quantify the dynamics of these settling particles, *Re_p_*, a critical parameter in fluid mechanics that measures the ratio of inertial forces to viscous forces acting on a particle in a fluid, was calculated. This calculation facilitates a better understanding of the settling behavior within the experimental setup, aligning with numerous studies in the literature that investigate particle dynamics in fluid media [67]. The *Re_p_* was calculated as follows:

(10) $${Re}_{p}=\frac{\rho_{f}V_{s}d_{e}}{\mu }$$

Here, *ρ_f_* is the fluid density (kg/m^3^), *V_s_* is the settling velocity (m/s), *d_e_* is the equivalent particle diameter (m). In this study, despite the particles having a sphericity of 0.7, we approximate *d_e_* as the mean particle diameter, assuming a roughly spherical shape for simplicity. The variable *μ* denotes the shear viscosity, measured in Pa⋅s.

The C_D*_ value for a sphere in a Newtonian fluid, which represents the standardized measure of drag acting on the sphere across various theoretical and flow conditions, was determined using a model [46,68]:

(11) $$C_{D*}=\frac{24}{{Re}_{p}}+\frac{2.6(\frac{{Re}_{p}}{5})}{1+{\left(\frac{{Re}_{p}}{5}\right)}^{1.52}}+\frac{0.411{\left(\frac{{Re}_{p}}{263000}\right)}^{-7.94}}{1+{\left(\frac{{Re}_{p}}{263000}\right)}^{-8}}+\frac{0.25\left(\frac{{Re}_{p}}{10^{6}}\right)}{1+\left(\frac{{Re}_{p}}{10^{6}}\right)}$$

To calculate the experimental *C_D_*, which measures the drag force experienced by a particle relative to the fluid flow around it during the experiments, we employed the following equation [69,70]:

(12) $$C_{D}=\frac{4\left(\rho_{p}-\rho_{f}\right)gd_{e}}{3\rho_{f}{V_{s}}^{2}}$$

For non-Newtonian fluids, particularly shear-thinning fluids, the behavior under different shear rates varies significantly from that of Newtonian fluids. Unlike Newtonian fluids, which exhibit a constant viscosity regardless of changes in shear rate, shear-thinning fluids demonstrate a decrease in viscosity as the shear rate increases [71,72,73]. To accurately model this characteristic, we used a modified equation to describe the dependence of viscosity on shear rate. This relationship enables the determination of shear viscosity based on the observed shear rate, as follows:

For *Re* < 2, the corresponding shear rate associated with the movement of proppants within the fluid was determined:

(13) $$\dot{\gamma }=\frac{V_{s}}{d_{e}}$$

Similarly, for the intermediate flow regime where 2 < *Re* < 500, the shear rate associated with the movement of proppants within the fluid was calculated [74]:

(14) $$\dot{\gamma }=\frac{{3V}_{s}}{d_{e}}$$

To further understand the viscoelastic properties of fluids and the extent of fluid deformation during proppant movement within FR solutions, it is crucial to evaluate the *W_i_*. *W_i_* is a measure of the ratio between elastic and viscous forces within the fluid [34,75]. Specifically, this dimensionless number is calculated by multiplying the fluid’s λ by the characteristic shear rate, which, in this context, is induced by the settling of a particle. A relatively low *W_i_* implies that the fluid exhibits negligible elastic effects in response to the deformations caused by moving particles.

(15) $$W_{i}=\frac{Elastic\;force}{Viscous\;force}=\lambda \dot{\gamma }$$

## Figures and Tables

**Table 1:** Experimental results: effects of Na_2_CO_3_ and NaOH doses on divalent cation removal, residual concentrations, and pH in FPW treatment at room temperature.

Treatment Conditions		Post-Treatment									
Molar Ratio of Na_2_CO_3_	C_NaOH_ (mol/L)	C_Na_ (ppm)	C_Ca_ (ppm)	E_Ca_ (%)	C_Mg_ (ppm)	E_Mg_ (%)	C_Fe_ (ppm)	E_Fe_ (%)	C_Sr_ (ppm)	E_Sr_ (%)	pH ± 0.10
0.8	0	80985	1836.7	85.97	623.1	27.71	0.35	99.1	752.1	57.32	7.24
1	0	84532	20.9	99.81	351.6	59.25	0	100	15.26	98.83	8.89
1.2	0	87884	0.6	100	169.2	80.38	0	100	0	100	10.02
1	0.0167	84358	3.8	99.97	31.6	96.33	0	100	0	100	9.57
1	0.0467	85414	3.0	99.97	1.05	99.88	0	100	0	100	10.01
1	0.0667	86011	1.2	99.99	0.5	99.94	0	100	0	100	10.81

**Table 2:** Curve-fitting results for FR solutions: flow consistency index, *K*, flow behavior index, *n*, and second constant viscosity, $\eta_{\infty },$ using power-law and Sisko models.

Samples	$K\;(Pa\cdot s^{n})$	n	$\eta_{\infty }\;(mPa\cdot s)$	*R* ^2^
1 gpt FR in DI water	0.2239	0.4403	N/A	0.998
2 gpt FR in DI water	0.3964	0.4279	N/A	0.998
3 gpt FR in DI water	0.6031	0.4371	N/A	0.999
1 gpt FR in UTFPW	0.0223	0.0001	2.5	0.999
2 gpt FR in UTFPW	0.0248	0.0008	2.9	0.999
3 gpt FR in UTFPW	0.0402	0.0155	3.2	0.999
1 gpt FR in TFPW	0.0272	0.2533	3.1	0.999
2 gpt FR in TFPW	0.0472	0.1152	3.7	0.999
3 gpt FR in TFPW	0.0664	0.0753	4.1	0.999

**Table 3:** Summary of relaxation time, λ, calculated from the inverse of the intersection point between storage modulus and loss modulus curves.

Samples	FR conc. (gpt)	λ (s)	Samples	FR conc. (gpt)	λ (s)	Samples	FR conc. (gpt)	λ (s)
DI water	1	10	UTFPW	1	0.12	TFPW	1	1.11
DI water	2	11.1	UTFPW	2	0.14	TFPW	2	2
DI water	3	15.4	UTFPW	3	0.25	TFPW	3	4.55

**Table 4:** Settling velocity of sand proppants with various size in FR solutions.

		Settling Velocity (cm/s)							
	Proppants	Size (16–30)	STD	Size (20–40)	STD	Size (30–50)	STD	Size (40–70)	STD
Samples									
DI 1gpt		0.680	0.207	0.244	0.020	0.145	0.003	0.065	0.031
DI 2gpt		0.216	0.048	0.111	0.037	0.028	0.009	0.015	0.005
DI 3gpt		0.139	0.045	0.075	0.024	0.014	0.002	0.004	0.000
UTFPW 1gpt		12.701	1.818	7.823	0.603	5.648	0.322	4.354	0.348
UTFPW 2gpt		11.728	1.065	7.601	0.631	3.982	0.397	3.790	0.355
UTFPW 3gpt		10.177	0.832	6.504	0.502	3.470	0.490	3.022	0.139
TFPW 1gpt		10.747	1.501	7.106	0.392	4.479	0.216	4.073	0.391
TFPW 2gpt		10.122	1.471	6.408	0.452	4.060	0.301	3.409	0.315
TFPW 3gpt		9.497	1.502	5.193	0.499	2.685	0.341	2.453	0.153

**Table 5:** Concentrations of cations and anions in brine produced from the Duvernay Formation.

Cations		Anions	
Ion	mg/L	Ion	mg/L
Na^+^	67,162	Cl^−^	145,700
K^+^	1949.9	Br^−^	1570
Ca^2+^	13,088	I^−^	120
Mg^2+^	861.9	HCO_3_^−^	40.7
Ba^2+^	17	SO_4_^2−^	276
Sr^2+^	1312	CO_3_^2−^	Nil
Fe^2+^	38.6	OH^−^	Nil

**Table 6:** Physical properties of sand proppant.

Proppants	16/30	20/40	30/50	40/70
Mean Particle Diameter (mm)	0.92	0.60	0.45	0.34
Bulk Density (g/cm^3^)	1.50	1.51	1.51	1.51
Specific Density (g/cm^3^)	2.52	2.56	2.65	2.61
Sphericity (Krumbein)	0.7	0.7	0.7	0.7

## Data Availability

The original contributions presented in this study are included in the article. Further inquiries can be directed at the corresponding author.
